# Killer immunoglobulin-like receptor genotype did not correlate with response to anti-PD-1 antibody treatment in a Japanese cohort

**DOI:** 10.1038/s41598-018-34044-z

**Published:** 2018-10-29

**Authors:** Yoshihiro Ishida, Chisa Nakashima, Hiroto Kojima, Hidenori Tanaka, Taku Fujimura, Shigeto Matsushita, Yuki Yamamoto, Koji Yoshino, Yasuhiro Fujisawa, Atsushi Otsuka, Kenji Kabashima

**Affiliations:** 1 Department of Dermatology, Graduate School of Medicine, Kyoto University, Kyoto, Japan; 2 HLA Foundation Laboratory, Kyoto, Japan; 3 Department of Dermatology, Tohoku University Graduate School of Medicine, Sendai, Japan; 4 Department of Dermato-Oncology/Dermatology, National Hospital Organization Kagoshima Medical Center, Kagoshima, Japan; 5 Department of Dermatology, Wakayama Medical University, Wakayama, Japan; 6 Department of Dermatology, Tokyo Metropolitan Cancer and Infectious Disease Center Komagome Hospital, Tokyo, Japan; 7 Department of Dermatology, University of Tsukuba, Tsukuba, Japan; 8 Translational Research Department for Skin and Brain Diseases, Kyoto University Graduate School of Medicine, 54 Shogoin-Kawara-cho, Sakyo-ku, Kyoto, Japan

## Abstract

Immune checkpoint blockade (ICB) induces a remarkable response in patients with certain cancers. However, the response rate is not yet satisfactory. Biomarkers that help physicians identify patients who would benefit from ICB need to be developed. Killer immunoglobulin-like receptors (KIRs) are a class of receptors that are mainly expressed by natural killer cells. KIR genotypes have been shown to influence the outcomes of patients with neuroblastoma and hematopoietic malignancies. KIRs may thus influence the clinical outcomes of melanoma patients receiving nivolumab. We aimed to identify the KIR genotype, or KIR/KIR-ligand combinations, which influence the outcomes of melanoma patients receiving nivolumab. We genotyped 112 melanoma patients who were treated with nivolumab for KIR and human leukocyte antigen. The clinical records of the patients were analyzed to determine if they showed a response to nivolumab, and whether or not they experienced adverse events. Our analysis showed that no KIR gene was associated with a response to nivolumab. The KIR/KIR-ligand combination did not correlate with a response to nivolumab. KIR genes were not predictive of experiencing adverse events of grade 2 or greater. We conclude that the KIR genotype or KIR/KIR-ligand genotype do not show predictive value in melanoma patients receiving nivolumab.

## Introduction

Although immune checkpoint blockade (ICB) has revolutionized the management of malignancies, there remain a considerable number of patients who do not respond. There is thus a great interest in developing predictive biomarkers to help identify patients who would benefit from ICB. These biomarkers include serum lactate dehydrogenase level^1^, baseline lymphocyte to neutrophil ratio^2^, PD-L1 expression of tumor cells^3^, mutational load^4^, gut microbiome^5^, and human leukocyte antigen (HLA) allele of the host^6,7^.

Recently, killer immunoglobulin-like receptor (KIR) genotypes have been shown to influence clinical outcomes in neuroblastoma patients receiving IL-2 immunotherapy or anti-GD1 antibody treatment^8,9^. KIRs are a class of regulatory molecules that are mainly expressed by natural killer (NK) cells. NK cells play a major role in tumor immunity, and their activation is regulated by a complex integration of inhibitory and stimulatory signals. Among the regulatory signals, KIRs are important for inter-individual difference in NK cell activity. KIR genes exhibit high allelic and haplotypic diversity, which results in variation in the NK cell repertoire among individuals. In several KIR genes, KIR-ligands are identified as HLA molecules. Examples of this include KIR1DL1/HLA-C1, KIR2DL1/HLA-C2, and KIR3DL1/HLA-Bw4. Absence/presence of the KIR/KIR–ligand pair adds further diversity to the individual immune profile^10^.

In light of the pressing need to develop biomarkers for ICB, we asked if the KIR or compound KIR–ligand genotype predicts the outcome of ICB by studying melanoma patients treated with nivolumab. We also asked if immune-related adverse events caused by ICB are associated with the KIR genotypes because they emulate autoimmune diseases, such as interstitial pneumonia, Hashimoto’s thyroiditis, and type 1 diabetes, some of which were shown to be influenced by KIR genotypes^11,12^. We conducted a genetic association study in which 112 melanoma patients receiving nivolumab therapies were genotyped for KIR and HLA. In this study, we report that the KIR or KIR–ligand genotype does not influence the clinical outcomes of melanoma patients receiving nivolumab therapy.

## Results

### No KIR genotype was associated with clinical response

One hundred and twelve melanoma patients were enrolled. Of these, 58 were male and 54 were female. The median age was 68 years old (26–93, minimum-maximum). Overall, 27.7% of patients (n = 31) showed a response to nivolumab therapy. Clinical response was not assessable in five patients, and they were removed in subsequent related analyses. Patient characteristics were similar between responders and non-responders except for disease subtype (Table 1). We noted that mucosal melanoma was under-represented in responders. Although not significant, the odds ratio of mucosal melanoma responding to the therapy was 0.684 (*p-*value: 0.058, 95% CI: 0.228–1.932) compared to the cutaneous subtype set as baseline. Melanoma subtypes were controlled for in the subsequent multivariate analysis. In both univariate and multivariate analysis, none of the KIR genes correlated with response to nivolumab (Table 2).

**Table 1:** Patient characteristics and clinical response.

	Responder (n = 31)		Non-responder (n = 76)	
Age - median (min - max)	68 (27–89)		66 (26–93)	
Sex				
Female	15		37	
Male	16		39	
RECIST category				
	CR	7	SD	19
	PR	17	PD	57
	dSD	7		
Disease subtypes				
Cutaneous	12 (26.1%)		34 (73.9%)	
Mucosal	7 (19.4%)		29 (80.6%)	
Acral	8 (44.4%)		10 (55.6%)	
Uveal	2 (40.0%)		3 (60%)	
Unknown	2 (100%)			

RECIST: response evaluation criteria in solid tumors, CR: complete response, PR: partial response, dSD: durable stable disease, SD: stable disease, PD: progressive disease.

**Table 2:** KIR genotype and clinical response.

KIR gene	KIR Present	KIR Absent	Univariate	Multivariate	*p*-value	Odds ratio (95% CI)
	Responders/total (rate)	Responders/total (rate)	*p*-value	Odds ratio (95% CI)		
2DL1	31/112 (0.28)	0/0 (NA)	NA	NA	0.36	NA
2DL2	6/14 (0.43)	25/98 (0.26)	0.23	2 (0.62–6.5)	0.26	2.4 (0.71–8.1)
2DL3	31/111 (0.28)	0/1 (0)	0.99	NA	0.15	NA
2DL4	31/112 (0.28)	0/0 (NA)	NA	NA	0.42	NA
2DL5	11/41 (0.27)	20/71 (0.28)	0.89	0.94 (0.39–2.2)	0.71	1.2 (0.46–3)
2DS1	11/39 (0.28)	20/73 (0.27)	0.9	1.1 (0.43–2.5)	0.73	1.3 (0.5–3.3)
2DS2	7/15 (0.47)	24/97 (0.25)	0.11	2.5 (0.79–7.6)	0.15	3.1 (0.96–10)
2DS4	31/108 (0.29)	0/4 (0)	0.99	NA	0.56	NA
2DS3	5/15 (0.33)	26/97 (0.27)	0.55	1.4 (0.41–4.6)	0.38	1.7 (0.46–5.6)
2DS5	7/30 (0.23)	24/82 (0.29)	0.5	0.72 (0.26–1.8)	0.37	0.85 (0.29–2.3)
2DS3/5	11/41 (0.27)	20/71 (0.28)	0.89	0.94 (0.39–2.2)	0.71	1.2 (0.46–3)
3DL1	31/108 (0.29)	0/4 (0)	0.99	NA	0.56	NA
3DS1	10/39 (0.26)	21/73 (0.29)	0.75	0.86 (0.35–2.1)	0.89	1.1 (0.4–2.7)
3DL2	31/112 (0.28)	0/0 (NA)	NA	NA	1	NA
3DL3	31/112 (0.28)	0/0 (NA)	NA	NA	0.99	NA

Presence of each KIR genotypes is analyzed for correlation with clinical response using logistic regression models. In multivariate analysis, melanoma subtypes and KIR genes are included in the model. NA denotes that the model did not converge.

### KIR/KIR-ligand combination was not associated with clinical response

Because KIR and HLA genes are independently inherited, a compound KIR-HLA genotype may be more predictive of the host’s immune profile, as shown previously^13^. First, we examined if the presence of matching HLA alleles was associated with clinical response. No KIR –HLA combinations were associated with clinical response to nivolumab (Table 3). Among the KIR genes tested, a compound HLA-Bw4 and KIR3DL1 genotype was well characterized. The expression level of KIR3DL1 was predicted by the allotype. HLA-Bw4 is further classified into low- and high-affinity allotypes. Combining these two variables, we predicted the interaction strength between KIR3DL1 and HLA-Bw4, as described previously^14^. However, the predicted interaction strength between KIR3DL1 and its ligands was not associated with clinical response (Table 4).

**Table 3:** KIR–KIR ligand interaction and clinical response.

HLA epitope/KIR gene	Interaction	No interaction	p-value	Odds ratio (95% CI)
	Responders/total (rate)	Responders/total (rate)		
HLA-Bw4/KIR3DL1	25/89 (0.28)	6/18 (0.33)	0.78	0.78 (0.25–2.4)
HLA-C1/KIR2DL2	6/13 (0.46)	25/94 (0.27)	0.19	2.3 (0.68–7.8)
HLA-C1/KIR2DL3	30/104 (0.29)	1/3 (0.33)	1.00	0.81 (0.062–24)
HLA-C2/KIR2DL1	6/15 (0.4)	25/92 (0.27)	0.36	1.8 (0.55–5.6)

All patients were assessed for presence of each KIR gene/KIR ligand pair. Fisher’s exact test was used to estimate *p*-values and odds ratio.

**Table 4:** Predicted interaction strength between KIR3DL1 and HLA-Bw4 and clinical response.

Interaction strength	Univariate	Multivariate	Odds ratio (95% CI)	p-value	Odds ratio (95% CI)
	Responders/total (rate)	p-value			
No	6/22 (0.27)	NA	NA	NA	NA
Weak	11/42 (0.26)	0.79	1.1 (0.51–2.6)	0.74	1.2 (0.5–2.9)
Strong	14/47 (0.3)	0.72	1.1 (0.55–2.4)	0.95	1 (0.48–2.2)

HLA-Bw4 epitopes are classified into high affinity, low affinity or absent. KIR3DL1 expression was estimated from the KIR allotypes. Strength of the interaction between KIR3DL1 and its ligand interaction was predicted based on these results and then was analyzed for correlation with clinical response. ‘No interaction’ group was set as baseline.

### KIR genotype was not associated with adverse events

Next, we asked if a particular KIR genotype influenced whether or not a patient developed adverse events. Overall, our cohort showed 35 adverse events of grade 2 or greater. Frequent adverse events included abnormal liver function (4 patients), colitis (4 patients), hypothyroidism (4 patients), interstitial pneumonia (4 patients), low platelet count (3 patients), and uveitis (3 patients). None of the adverse effect categories had a sufficient number of patients to be analyzed meaningfully. We analyzed if a patient developed any “significant” adverse events, defined as adverse events of grade 2 or greater. Individuals with KIR2DL2 seem to be more likely to experience adverse events, with a crude *p*-value of 0.0461 and an odds ratio of 3.4 (95% CI: 1.04–11.1) (Table 5). We then adjusted the *p-*values using the Benjamin-Hochberg procedure. The adjusted *p*-value for KIR2DL2 was 0.34.

**Table 5:** Correlation of KIR genes with adverse events grade 2 or greater.

KIR gene	KIR gene present	KIR gene absent			
	Patients with adverse events/total (rate)	Patients with adverse events/total (rate)	p-value (crude)	p-value (adjusted)	Odds ratio (95% CI)
2DL1	29/112 (0.26)	0/0 (NA)	NA	NA	NA
2DL2	7/14 (0.5)	22/98 (0.22)	0.046*	0.34	3.4 (1–11)
2DL3	28/111 (0.25)	1/1 (1)	0.26	0.70	0 (0–6.6)
2DL4	29/112 (0.26)	0/0 (NA)	NA	NA	NA
2DL5	10/41 (0.24)	19/71 (0.27)	0.83	0.83	0.88 (0.35– 2.2)
2DS1	8/39 (0.21)	21/73 (0.29)	0.38	0.70	0.64 (0.25–1.6)
2DS2	7/15 (0.47)	22/97 (0.23)	0.061	0.34	2.9 (0.92–9.9)
2DS4	29/108 (0.27)	0/4 (0)	0.57	0.70	Inf (0.31–Inf)
2DS3	5/15 (0.33)	24/97 (0.25)	0.53	0.70	1.5 (0.46–4.8)
2DS5	6/30 (0.2)	23/82 (0.28)	0.47	0.70	0.64 (0.23–1.8)
2DS3/5	10/41 (0.24)	19/71 (0.27)	0.83	0.83	0.88 (0.35–2.2)
3DL1	29/108 (0.27)	0/4 (0)	0.57	0.70	Inf (0.31–Inf)
3DS1	8/39 (0.21)	21/73 (0.29)	0.38	0.70	0.64 (0.25–1.6)
3DL2	29/112 (0.26)	0/0 (NA)	NA	NA	NA
3DL3	29/112 (0.26)	0/0 (NA)	NA	NA	NA

Presence of each KIR gene is analyzed for correlation with developing adverse events CTCAE grade 2 or greater. The table shows both crude p-values and p-valued adjusted using Benjamini-Hochberg procedure. * < 0.05, NA: value was not meaningfully calculated, Inf: infinite number.

## Discussion

Despite the recognized role of NK cells in cancer immunology, our study does not demonstrate that the KIR genotype correlates with clinical outcomes in nivolumab therapy. A previous study showed that the KIR genotype was associated with the development, progression, and metastasis of melanoma^15^. Our study, however, suggests that KIR is not a major determinant in discriminating responders from non-responders in melanoma patients receiving anti-PD-1 therapy. In our cohort, the KIR genotype does not appear to affect whether or not a patient experiences adverse events.

Our results are inconclusive in terms of whether the action of anti-PD-1 therapy is dependent on NK cells. The action of anti-PD-1 therapy may depend solely on CD8+ T cells and not on NK cells. Mounting evidence, however, shows that NK cells play a part in regulating the tumor microenvironment. One recent report showed that, in melanoma patients, NK cells recruit conventional DCs to the tumor microenvironment and augment anti-tumor immunity^16^. It is more likely that NK cells play a role in tumor immunity, but the success of anti-PD-1 therapy is dependent on factors other than KIRs. We speculate that the KIR genotype may prove important for melanoma patients in different contexts, and that the KIR genotype may affect the outcome of anti-CTLA4 treatment or NK cell adoptive transfer therapies. KIR genotyping in these contexts would merit consideration.

Predicting adverse events caused by ICB is of great interest. However, our research results show that the KIR genotype does not have predictive value in respect to developing adverse events. In our dataset, KIR2DL2 appeared to confer a risk of developing adverse events. Correction for multiple testing declined the significance of KIR2DL2. Previous reports on predicting adverse events include the clonal expansion of CD8+ cells in circulation^17^, or the diversification of the T-cell repertoire^18^. It is likely that CD8+ T cells, but not NK cells, mediate the adverse events caused by ICB.

There are several limitations in this study. First, patients were enrolled from regular clinical practices from multiple hospitals. This study design precludes the uniformity of study participants and evaluation criteria. For example, our ad hoc analysis showed considerable variation in patient characteristics among participating hospitals. A multivariate analysis controlling for hospitals did not show any correlation between KIR genotype and clinical outcomes (data not shown). Nevertheless, we cannot confidently exclude the possibility that there may be unmeasurable biases at play in this study group.

Second, our study is confined to the Japanese population. KIR genotypes are highly variable among ethnic groups^19^. One cannot necessarily assert that our findings would apply to other countries or ethnic groups. Moreover, acral/mucosal melanoma is overrepresented in Japan as compared to Western countries, where cutaneous melanomas predominate^20^. Such discrepancy in melanoma subtypes may further complicate the interpretation of our results. Considering the total absence of possible associations in our study, however, the chance of identifying a clinically relevant KIR genotype in other ethnic groups would be slim.

Third, adverse events grade 2 or greater are documented only in 35 cases out of 112 patients analyzed. Our study may be underpowered to conclusively decline associations between KIR genotypes and adverse events.

Lastly, our study is focused solely on KIR and KIR-HLA compound genotypes. This study design overlooks the possibilities that polymorphisms in other genes may influence the response to cancer immunotherapy in melanoma patients.

In conclusion, our genetic association study did not detect an association between KIR genotype and clinical outcomes of nivolumab treatment in a Japanese cohort. Further studies on KIR genotype and cancer should focus on other treatment modalities, such as anti-CTLA4 treatment or NK cell therapy.

## Methods

### Patients

We enrolled melanoma patients who were treated with nivolumab at participating hospitals in Japan between July 2014 and October 2017. Patients who had follow-up periods less than 3 months after the initiation of nivolumab treatment were excluded from the study. This study was approved by the institutional review board at Kyoto university (G1014). Written informed consent was obtained from all participants. The study was carried out according to the approved protocol.

### HLA and KIR genotyping

DNA was extracted from the patients’ blood using a QuickGene DNA whole blood kit S (Kurabo, Osaka, Japan). Genotyping was performed at HLA Laboratory (Kyoto, Japan). Briefly, a DNA library was prepared using IGS-HLA version 4.0 and IGS-KIR version 2.1 typing kits (Scisco Genetics, Inc., Seattle, WA) according to the manufacturer’s protocol. The library was sequenced on the MiSeq platform (Illumina, San Diego, CA).

### KIR–ligand analysis

In analyzing the interaction between the HLA allele and KIR genes, each patient was evaluated for KIR3DL1, KIR2DL1, and KIR2DL2/3. A patient was considered to carry a KIR gene if the patient had at least one copy of the gene. Next, using a table extracted from Marra *et al*.^13^, HLA alleles were assigned to KIR-ligands, and then the absence or presence of KIR–ligand combination was determined.

The interaction between HLA-B alleles and KIR3DL1 allelic polymorphism was analyzed according to the previously described procedure^9^. Briefly, HLA-B alleles were assigned to HLA-Bw4-80I, HLA-Bw4-80T, or Bw6 using the immune polymorphism database^21^, and then each patient was categorized according to compound epitopes. KIR3DL1 alleles were classified into functional subtypes according to the previous publication^22^. Compound HLA-B epitope and KIR3Dl1 subtypes were combined to predict the strength of interaction between KIR3DL1 and HLA-B ligands.

### Statistical analysis

We used response evaluation criteria in solid tumors (RECIST) 1.1 to assess clinical response. Response was defined as complete response, partial response, or stable disease lasting more than 6 months. We used common terminology criteria for adverse events (CTCAE) version 4.0 to evaluate adverse events. Significant adverse events were defined as those of grade 2 or greater. The logistic regression model was used to estimate the odds ratio and 95% confidence interval (CI) in evaluating the association of KIR genotypes and KIR–ligand combination with clinical response. In multivariate analysis, age, sex, and tumor subtypes were initially screened for potential association with clinical outcomes, and those with significant associations were then used in the final analysis. Fisher’s exact test was used in the remaining analyses. Considering the exploratory nature of this study, correction for multiple testing was not performed initially. When an analysis yielded a *p*-value smaller than 0.05, adjusted *p*-values were computed using the Benjamin-Hochberg procedure. A *p*-value < 0.05 was considered statistically significant. All statistical analyses were carried out with R software (version 3.4.3, R core team, Vienna, Austria). Two-sided Fisher’s exact test was performed as instructed in the exact2x2 package^23^.

The datasets generated from the current study is included in the article and its Supplementary Information Material.

## Electronic supplementary material

Dataset 1
